# Supplementary material for: Non-cognitive skills and labour market performance of immigrants
Source: PLoS One. 2023 May 3;18(5):e0281048. doi: 10.1371/journal.pone.0281048 (PMC10155982; doi:10.1371/journal.pone.0281048)
Supplement: S1 File — (PDF) [file pone.0281048.s001.pdf]

Appendices

Table A1. Big-5 Personality Inventory

I see myself as someone who...
Q1. ...does a thorough job
Q2. ...is communicative, talkative
Q3. ...is sometimes some what rude to others
Q4. ...is original, comes up with new ideas
Q5. ...worries a lot
Q6. ...has a forgiving nature
Q7. ...tends to be lazy
Q8. ...is outgoing, sociable
Q9....values artistic experiences
Q10. ...gets nervous easily
Q11. ...does things effectively and efficiently
Q12. ...is reserved
Q13. ...is considerate and kind to others
Q14. ...has an active imagination
Q15. ...is relaxed, handle stress well
The question prompted to respondents is: "Here indicate how closely you agree with the statement by checking one of the boxes on the scale between 1 to 7. 1 means you completely disagree, and 7 means you completely agree. If your views fall somewhere in between, you can choose any number in between."
The Big-5 measures are calculated as follows: Extraversion: Q2 + Q8 + Reversed(Q12); Aggreable: Q3 + Q6 + Reversed(Q13); Conscientiousness: Q1 + Reverseds(Q7) + Q11; Neuroticm: Q5 + Q10 + Reversed(Q15); Openness to experience: Q4 + Q9 + Q14;

Table A2. Descriptive Statistics.

	Natives	Immigrants	Immigrants from EU Countries	Immigrants from Non-EU Countries	Immigrants from Guest Worker C
Employment status (=1)	0.752	0.681	0.724	0.636	0.654
	(0.432)	(0.466)	(0.447)	(0.481)	(0.476)
Extraversion	14.577	13.880	14.206	13.543	13.787
	(3.358)	(3.533)	(3.442)	(3.594)	(3.538)
Emotional stability (reversed neuroticism)	12.142	11.812	11.745	11.881	11.638
	(3.626)	(3.634)	(3.736)	(3.524)	(3.619)
Conscientiousness	17.746	17.899	18.011	17.782	17.694
	(2.725)	(2.778)	(2.71)	(2.842)	(2.866)
Openness to experience	13.578	12.797	13.064	12.523	12.227
	(3.502)	(3.862)	(3.824)	(3.881)	(3.891)
Agreeableness	16.230	16.676	16.517	16.841	16.505
	(2.879)	(3.032)	(2.898)	(3.155)	(3.007)
Years since migration		22.579	24.847	20.237	26.048
		(11.055)	(11.256)	(10.334)	(10.015)
Age	42.975	43.070	45.090	40.985	43.536
	(12.775)	(12.14)	(11.866)	(12.066)	(11.812)
Age of arrival to Germany		20.498	20.248	20.757	17.499
		(11.653)	(11.138)	(12.157)	(9.538)
Female (=1)	0.534	0.544	0.551	0.536	0.510
	(0.499)	(0.498)	(0.497)	(0.499)	(0.5)
Married (=1)	0.607	0.772	0.742	0.804	0.827
	(0.488)	(0.419)	(0.438)	(0.397)	(0.379)
Family abroad	0.014	0.203	0.208	0.198	0.126
	(0.116)	(0.402)	(0.406)	(0.398)	(0.331)
Spouse abroad		0.00266	0.00434	0.00092	0.00518
		(0.051)	(0.066)	(0.03)	(0.072)
Refugee		0.248	0.198	0.300	0.032
		(0.432)	(0.398)	(0.458)	(0.176)
Remittances (Euros)		453.367	580.360	322.276	458.224
		(2279.532)	(2844.248)	(1475.096)	(2460.622)
Years of Education	12.206	10.605	10.750	10.455	9.599
	(2.69)	(2.535)	(2.584)	(2.475)	(2.081)
Current health-bad (=1)	0.024	0.035	0.037	0.032	0.044
	(0.154)	(0.183)	(0.189)	(0.177)	(0.205)
Current health-poor (=1)	0.111	0.139	0.140	0.138	0.160
	(0.314)	(0.346)	(0.347)	(0.344)	(0.367)
Current health-satisfactory (=1)	0.317	0.300	0.308	0.293	0.287
	(0.465)	(0.458)	(0.462)	(0.455)	(0.452)
Current health-good (=1)	0.448	0.426	0.415	0.437	0.421
	(0.497)	(0.494)	(0.493)	(0.496)	(0.494)
Current health-very good (=1)	0.101	0.100	0.101	0.100	0.089
	(0.301)	(0.3)	(0.301)	(0.3)	(0.285)
Household size	2.811	3.391	3.100	3.691	3.599
	(1.223)	(1.548)	(1.365)	(1.663)	(1.623)
Number of kids	0.623	1.017	0.790	1.252	1.125
	(0.926)	(1.21)	(1.06)	(1.307)	(1.214)
Capital income (Euros)	2281.873	1845.748	2402.983	1270.525	1346.627
	(13925.623)	(50207.652)	(68489.669)	(16740.79)	(12423.479)
#Observations	208,064	28,582	14,518	14,064	14,673

Note: Authors' own calculations from the SOEP (1984-2013).

Table A3. Descriptive Statistics by Country of Origin.

			Big-5 Personality Chracteristi[illegible]			
	#Observations	Employment probability	Extroversion	Emotional Stability	Conscientiousness	Ope[illegible]
Turkey	6,763	0.571	13.298	11.622	17.343	
		(0.495)	(3.55)	(3.465)	(2.97)	
Poland	3,107	0.756	14.134	11.498	18.079	
		(0.429)	(3.399)	(3.656)	(2.714)	
Former-Yugoslavia	2,836	0.695	13.865	11.397	18.030	
		(0.46)	(3.462)	(3.889)	(2.705)	
Italy	2,431	0.742	14.682	11.741	17.898	
		(0.438)	(3.309)	(3.688)	(2.904)	
Russia	2,224	0.716	13.443	12.231	18.514	
		(0.451)	(3.79)	(3.628)	(2.53)	
Kazakhstan	2,201	0.738	13.738	11.755	18.502	
		(0.44)	(3.291)	(3.218)	(2.245)	
Greece	1,180	0.768	13.821	11.591	17.801	
		(0.422)	(4.048)	(4.036)	(2.69)	
Romania	1,063	0.710	14.262	12.111	18.247	
		(0.454)	(3.25)	(3.963)	(2.789)	
Spain	620	0.742	14.377	11.700	18.682	
		(0.438)	(3.031)	(3.505)	(2.162)	
Austria	441	0.687	14.222	13.760	17.853	
		(0.464)	(3.376)	(3.528)	(2.517)	
Ukraine	333	0.492	13.399	12.105	17.982	
		(0.501)	(3.185)	(3.08)	(2.298)	
France	255	0.725	0.725	0.725	0.725	
		(0.447)	(2.776)	(3.354)	(2.683)	
USA	247	0.789	15.988	12.725	17.174	
		(0.409)	(3.112)	(3.212)	(3.271)	
Holland	244	0.656	15.918	13.209	17.852	
		(0.476)	(3.043)	(2.744)	(2.443)	
Croatia	243	0.588	14.259	12.617	17.671	
		(0.493)	(2.942)	(1.945)	(3.097)	
Czech Republic	217	0.719	13.968	10.622	17.862	
		(0.451)	(3.939)	(3.631)	(2.689)	
UK	212	0.759	13.991	11.439	17.986	
		(0.428)	(4.021)	(4.3)	(2.427)	
Hungary	198	0.758	14.818	12.404	18.641	
		(0.43)	(3.146)	(4.049)	(1.833)	
Iran	190	0.542	11.963	11.642	18.237	
		(0.5)	(3.914)	(3.238)	(2.36)	
Philippines	168	0.607	14.798	12.292	16.571	
		(0.49)	(2.697)	(3.169)	(4.043)	
Tajikistan	162	0.747	11.457	12.099	17.475	
		(0.436)	(4.299)	(4.044)	(2.367)	
Bosnia-Herzegovina	161	0.801	14.099	12.621	17.242	
		(0.4)	(3.669)	(3.703)	(2.522)	
Kyrgyzstan	125	0.856	12.376	11.872	17.624	
		(0.353)	(2.996)	(3.894)	(3.197)	
Others	2,961	0.657	14.486	12.499	17.872	
		(0.475)	(3.335)	(3.677)	(2.871)	

Note: Authors' own calculations from the SOEP (1984-2013).

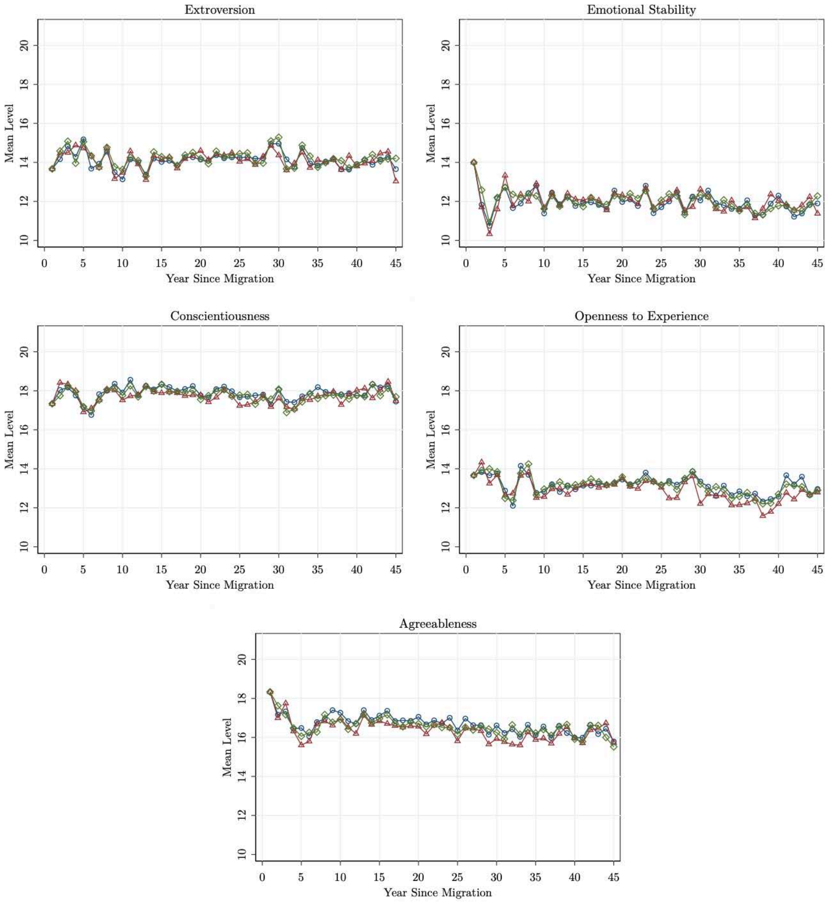

Fig A1. Behavior of Personality Characteristics over Years Since Migration Authors' own calculations from the SOEP for waves 2005 (circle), 2009 (triangle), and 2013 (diamond). The vertical axes give the mean level of each personality characteristic for each year since migration.

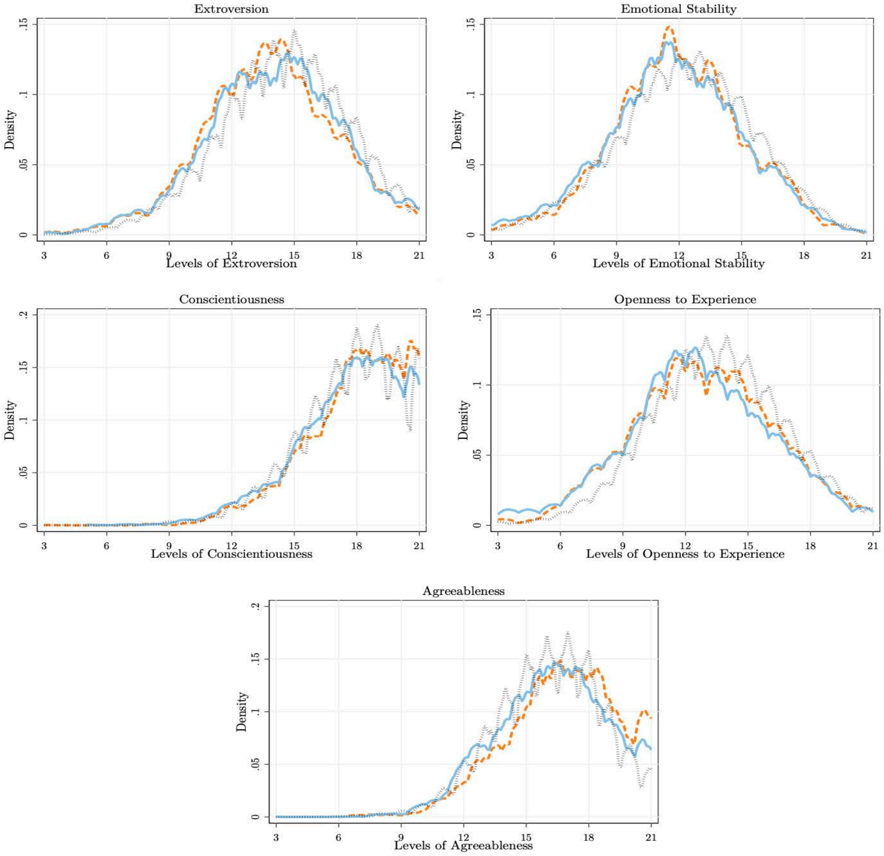

Fig A2. Distributions of Personality Characteristics by Immigrants and Natives
Authors' own calculations from the SOEP for waves 2005, 2009, and 2013. The figures show the kernel density estimates. Orange curves show the distributions among immigrants who have been in Germany for less than the median (20 years) and blue curves show the distribution among immigrants who have been in Germany longer than or equal to the median. The dotted black plots are the kernel density estimates for the natives.

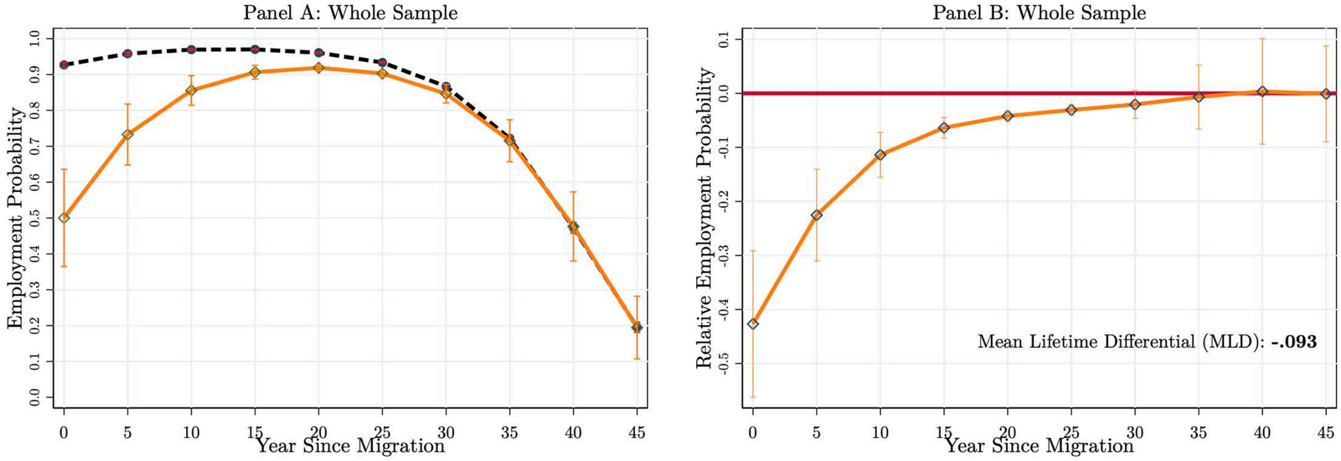

Fig A3. Sensitivity Checks of Benchmark Results: Pooled Probit Model
Authors' own calculations from SOEP (1984-2013). The figures are obtained by predicting the expected probability of employment using the parameter estimates of the pooled probit model for both immigrants and natives. The model specification allows for the full set of observed characteristics for both immigrants and natives (see Table 1). See the note under Figure 2 for further information.

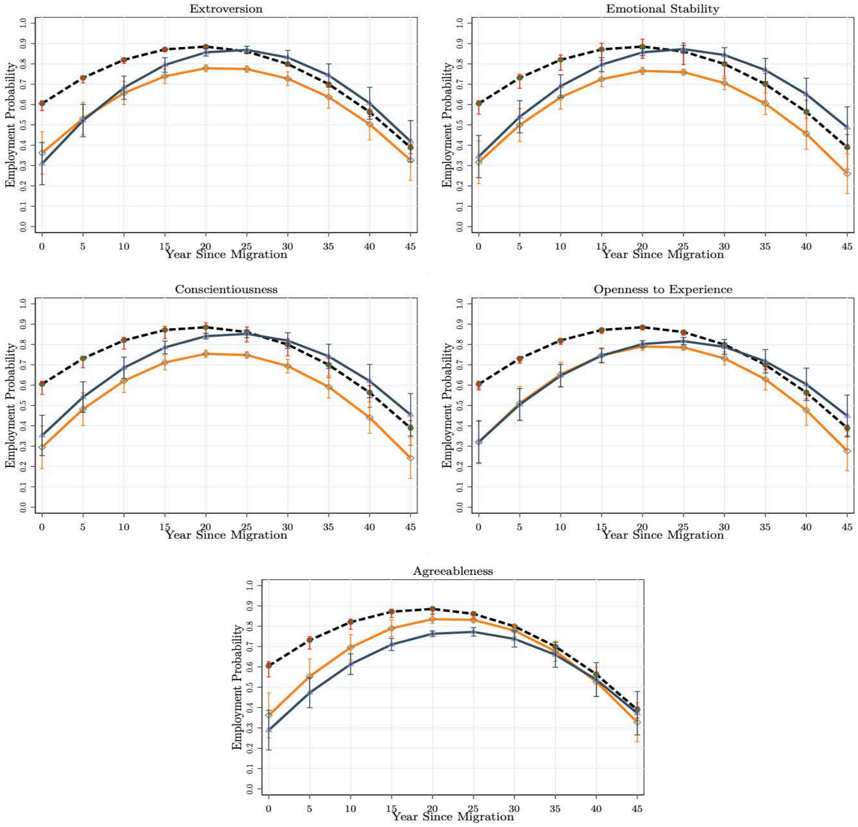

Fig A4. Comparing Immigrants and Average Natives: Life-Cycle Profiles
Authors' own calculations from the SOEP (1984-2013). The predicted employment probabilities of natives are given in the dashed black curve while immigrants' predicted employment probabilities are given for the low levels (orange curves with diamonds) and high levels (dark blue curves with triangles). The models are estimated with the correlated random-effects linear probability models (equations (1) and (2)) with the full set of characteristics. We use the delta method to calculate the standard errors. The 90% confidence intervals are represented as vertical lines.

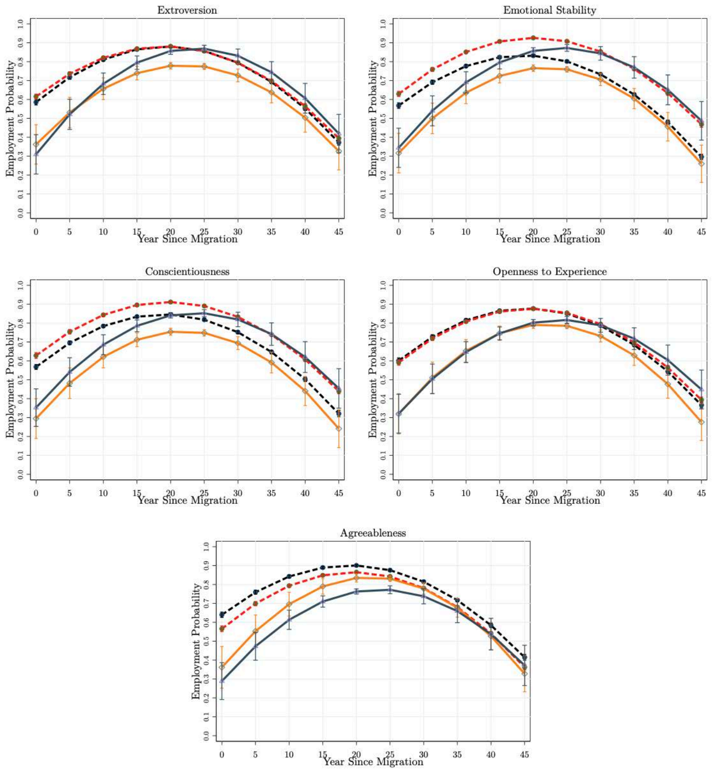

Fig A5. Comparing Immigrants and Natives with the Same Non-Cognitive Skill Types and Levels: Life-Cycle Profiles
Authors' own calculations from the SOEP (1984-2013). The predicted employment probabilities of natives are given in the dashed black curve while immigrants' predicted employment probabilities are given for the low levels (orange curves with diamonds) and high levels (dark blue curves with triangles). The models are estimated with the correlated random-effects linear probability models (equations (1) and (2)) with the full set of characteristics. We use the delta method to calculate the standard errors. The 90% confidence intervals are represented as vertical lines.

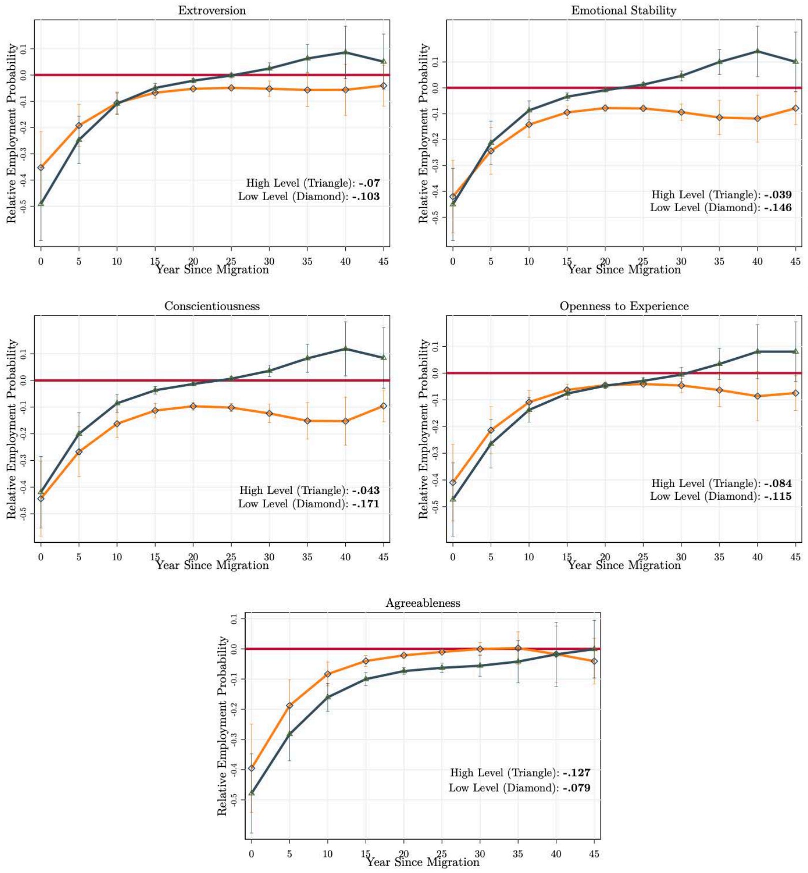

Fig A6. Comparing Immigrants and Natives with the Same Non-Cognitive Skill Types and Levels: Life-Cycle Profiles
Authors' own calculations from the SOEP(1984-2013). The model are estimated using pooled probit specification. The predicted employment probabilities of natives are given in the dashed black curve while immigrants' predicted employment probabilities are given in the smooth orange curve. We use the delta method to calculate the standard errors. The 90% confidence intervals are represented as vertical lines.
