# Supplementary material for: Non-cognitive skills and labour market performance of immigrants
Source: PLoS One. 2023 May 3;18(5):e0281048. doi: 10.1371/journal.pone.0281048 (PMC10155982; doi:10.1371/journal.pone.0281048)
Supplement: Attachment — Submitted filename: Editorial Letter and Response to Reviewers.pdf [file pone.0281048.s002.pdf]

Ms. Ref. No.: PONE-D-22-25219
Title: Non-Cognitive Skills and Labour Market Performance of Immigrants
PLOSONE

Dear Editor,

Thank you very much for your positive assessment of our paper and for giving us the opportunity to revise it. The insightful report we received from a reviewer helped us improve our paper. Below, we summarize what we consider to be the main issues raised by the reviewer and briefly describe how we have revised the paper in response. More detailed discussions can be found in the response letters to the reviewer.

The changes in the paper can be summarized as follows. First, there is no change in the results reported in the paper. The tables and programming codes are all intact. Second, we have updated the paper about an important conceptual issue related to “cognitive skills” and how they are related to overall “human capital”. In the first draft, we used the term cognitive skills synonymous with human capital acquired through formal education and training. Yet, it might create confusion. We have now updated the manuscript to prevent this. Instead of “cognitive skills”, we use “years of education”, the standard proxy for human capital. Second, we have explained why we have focused on the employment outcome. Finally, the paper is checked for grammar and typos. We have supplied two versions of the revision. In the first one, we have highlighted the changes in the paper using bold text (as there is no track changes in Latex). The second one gives a 'clean' version of the revision (without track changes). We are unsure about whether we should locate the paper in the PLOSONE Latex template. Yet, we are happy to do that quickly whenever it is asked.

We hope that you will find our revision satisfactory, and that the new version of the paper will be accepted for publication in the PLOSONE. If not, we are, of course, willing to make further changes.

We look forward to hearing from you in due course.
Sincerely,
Alpaslan Akay and Levent Yilmaz

Editors Comments and Requirements of the Journal

In the following, we answer to the requirements of the journal asked in the editorial email. The text in the email is reproduced in italic and our answers are coloured in blue. Please let us know what we have missed. We are happy to supply further information and materials

Dear Dr. Akay,

Thank you for submitting your manuscript to PLOS ONE. After careful consideration, we feel that it has merit but does not fully meet PLOS ONE's publication criteria as it currently stands. Therefore, we invite you to submit a revised version of the manuscript that addresses the points raised during the review process.

Please submit your revised manuscript by Dec 16 2022 11:59PM. If you will need more time than this to complete your revisions, please reply to this message or contact the journal office at plosone@plos.org. When you're ready to submit your revision, log on to https://www.editorialmanager.com/pone/ and select the 'Submissions Needing Revision' folder to locate your manuscript file.

Thank you very much for your positive assessment of our paper and giving us the opportunity to revise it. We have checked the webpage and we could not find any attached file for additional referees. We have replied to the comments of Referee #1 below.

Please include the following items when submitting your revised manuscript:

A rebuttal letter that responds to each point raised by the academic editor and reviewer(s). You should upload this letter as a separate file labeled 'Response to Reviewers'.

We have submitted the 'Response to Reviewers' file.

A marked-up copy of your manuscript that highlights changes made to the original version. You should upload this as a separate file labeled 'Revised Manuscript with Track Changes'.

We use Latex. We have highlighted the changes with the bold text. As you will see there are not many changes in the main text. The file is supplied as a PDF file. The Latex version is also available.

An unmarked version of your revised paper without tracked changes. You should upload this as a separate file labeled 'Manuscript'.

We have attached the clean version of the revised manuscript.

If you would like to make changes to your financial disclosure, please include your updated statement in your cover letter. Guidelines for resubmitting your figure files are available below the reviewer comments at the end of this letter.

There is no change. The University of Gothenburg has a special agreement with the journal, and they are going to pay promptly.

If applicable, we recommend that you deposit your laboratory protocols in protocols.io to enhance the reproducibility of your results. Protocols.io assigns your protocol its own identifier (DOI) so that it can be cited independently in the future. For instructions see: https://journals.plos.org/plosone/s/submission-guidelines#loc-laboratory-protocols. Additionally, PLOS ONE offers an option for publishing peer-reviewed Lab Protocol articles, which describe protocols hosted on protocols.io. Read more information on sharing protocols at https://plos.org/protocols?utm_medium=editorial-email&utm_source=authorletters&utm_campaign=protocols.

There is no experimental data used in the study.

***PLOS ONE* Journal Requirements:**

When submitting your revision, we need you to address these additional requirements.

1. Please ensure that your manuscript meets PLOS ONE's style requirements, including those for file naming. The PLOS ONE style templates can be found at

https://journals.plos.org/plosone/s/file?id=wjVg/PLOSOne_formatting_sample_main _body.pdf and
https://journals.plos.org/plosone/s/file?id=ba62/PLOSOne_formatting_sample_title_a uthors_affiliations.pdf

We have submitted in a standard Latex PDF format. We are happy to make detailed changes during the revision.

2. Please update your submission to use the PLOS LaTeX template. The template and more information on our requirements for LaTeX submissions can be found at http://journals.plos.org/plosone/s/latex.

We are unclear about whether we should locate the paper in the PLOS LaTeX template in this stage of the revision. We thought that we can do that later when the final version of the paper is accepted for publication.

3. *Thank you for stating the following financial disclosure:*

"No"

At this time, please address the following queries:

a) Please clarify the sources of funding (financial or material support) for your study. List the grants or organizations that supported your study, including funding received from your institution.

There is no specific funding used for this study. The publication fee is paid by the University of Gothenburg. We add the phrase “We appreciate the financial support from the University of Gothenburg” in the paper.

b) State what role the funders took in the study. If the funders had no role in your study, please state: “The funders had no role in study design, data collection and analysis, decision to publish, or preparation of the manuscript.”

The following phrase is added to the paper: “The funders had no role in study design, data collection and analysis, decision to publish, or preparation of the manuscript.”

c) If any authors received a salary from any of your funders, please state which authors and which funders.

There is no additional funding. I work in the University of Gothenburg, and it pays the publication fee.

d) If you did not receive any funding for this study, please state: “The authors received no specific funding for this work.”

We declare that “The authors received no specific funding for this work.”

Please include your amended statements within your cover letter; we will change the online submission form on your behalf.

We have written a cover latter for the revision and the statement is added therein.

4. Thank you for stating the following in your Competing Interests section:

"There is no conflict of interest on the study."

Please complete your Competing Interests on the online submission form to state any Competing Interests. If you have no competing interests, please state ""The authors have declared that no competing interests exist."", as detailed online in our guide for authors at http://journals.plos.org/plosone/s/submit-now
This information should be included in your cover letter; we will change the online submission form on your behalf.

It is stated.

5. *In your Data Availability statement, you have not specified where the minimal data set underlying the results described in your manuscript can be found. PLOS defines a study's*

minimal data set as the underlying data used to reach the conclusions drawn in the manuscript and any additional data required to replicate the reported study findings in their entirety. All PLOS journals require that the minimal data set be made fully available. For more information about our data policy, please see http://journals.plos.org/plosone/s/data-availability.

Upon re-submitting your revised manuscript, please upload your study's minimal underlying data set as either Supporting Information files or to a stable, public repository and include the relevant URLs, DOIs, or accession numbers within your revised cover letter. For a list of acceptable repositories, please see http://journals.plos.org/plosone/s/data-availability#loc-recommended-repositories. Any potentially identifying patient information must be fully anonymized.

Important: If there are ethical or legal restrictions to sharing your data publicly, please explain these restrictions in detail. Please see our guidelines for more information on what we consider unacceptable restrictions to publicly sharing data: http://journals.plos.org/plosone/s/data-availability#loc-unacceptable-data-access-restrictions. Note that it is not acceptable for the authors to be the sole named individuals responsible for ensuring data access.

We will update your Data Availability statement to reflect the information you provide in your cover letter.

The dataset (German Socio-Economic Panel Data) used in the paper is confidential, obtained from DIW (Berlin, Germany). The data can be obtained from DIW by anyone after assigning the confidential agreement. We can only supply data with their permission.

6. We note that you have stated that you will provide repository information for your data at acceptance. Should your manuscript be accepted for publication, we will hold it until you provide the relevant accession numbers or DOIs necessary to access your data. If you wish to make changes to your Data Availability statement, please describe these changes in your cover letter and we will update your Data Availability statement to reflect the information you provide.

There is no specific change now in the data availability statement. We can supply the data and information about the data as much as the confidentially rules allow us. For the SOEP, in particular the regional data, are confidential and they can be provided to third parties only with the permission of the company collects it (see www.diw.de)

7. PLOS requires an ORCID iD for the corresponding author in Editorial Manager on papers submitted after December 6th, 2016. Please ensure that you have an ORCID iD and that it is validated in Editorial Manager. To do this, go to 'Update my Information' (in the upper left-hand corner of the main menu), and click on the Fetch/Validate link next to the ORCID field. This will take you to the ORCID site and allow you to create a new iD or authenticate a pre-existing iD in Editorial Manager. Please see the following video for instructions on linking an ORCID iD to your Editorial Manager account: https://www.youtube.com/watch?v=_xcclfuvtxQ

The corresponding author is Alpaslan Akay. His ORCID ID is updated on the journal's page. His ORCID ID is 0000-0002-8543-9607. The ORCID ID of Levent Yilmaz is 0000-0002-5077-0877.

8. Please include your full ethics statement in the 'Methods' section of your manuscript file. In your statement, please include the full name of the IRB or ethics committee who approved or waived your study, as well as whether or not you obtained informed written or verbal consent. If consent was waived for your study, please include this information in your statement as well.

There is no specific ethical committee report is needed for the study. The data used is SOEP which is a survey data. The details can be obtained from www.diw.de.

Responses to Referee #1

Thank you very much for your careful reading of our paper and your constructive comments and suggestions. We have addressed each of them, and we believe that the paper has improved a lot as a result. We should highlight that the main text of the paper is not substantially rewritten. We have clarified the text in several places and expressed the motivation behind the arguments discussed. We appreciate the conceptual comment about the "cognitive skills". To prevent potential misunderstandings, we have updated the text (they are all given in bold text in the manuscript). We no longer stress education as a cognitive skill; we mention it as the standard human capital measure used in previous literature. The tables are not changed. We hope that you find the current version of the paper satisfactory, and, of course, we are happy to make further changes if you think it is necessary. Below, your original comments are reproduced in italics, and we have responded in a regular font in blue colour below each comment.

(1) Abstract: "This paper investigates how non-cognitive skills, e.g., memory, empathy, attention, imagination, and social skills...". Memory is not one of the non-cognitive skills. It's usually considered as a part of cognitive ability (fluid intelligence, in particular). The paper does not use direct measures of "empathy, attention, imagination, and social skills". I suggest replacing part with Big-5 Personality traits.

We thank to referee for this conceptual point. The referee is right. We are sorry about the mistake. We have taken out "memory". It is also correct that the paper does not use direct measures of "empathy, attention, imagination, and social skills". We have replaced them with Big-5 personality characteristics. We have updated it following the comment of the referee. Please see the new abstract.

(2) The paper examines the impact of non-cognitive skills on labor market performance. However, the only measure of the latter is employment. It is not clear why labor force participation and earnings are not examined.

This is an important point as well. There are several labour market outcomes, each of which can be investigated with a particular focus. As we mention in the conclusion, the

paper makes a comprehensive focus on one of the important outcomes and demonstrates how non-cognitive skills relate to labour market performance. Yet, the study could also cover earnings, health, and well-being outcomes by simply studying those as dependent variables. Instead, we mention this in the paper as future research to encourage researchers to focus on those outcomes. We also note that there is extensive literature investigating the relationship between non-cognitive skills and "employment" outcomes, which is larger than other outcomes. We aimed to take advantage of this extensive literature to enhance the discussions in the paper. Having said this, we have also experimented with, for instance, earnings. Among the unreported results, we have experimented with other outcomes such as *labour income*. The patterns are highly similar to those reported in the paper, as expected. We prefer leaving those analyses for future research as comprehensively focusing on each of them requires a full-length paper.

(3) I am a bit concerned about the use of education as measuring your cognitive skills. Education is not only related to cognitive skills but also non-cognitive skills. As Heckman and Rubenstein (2001) and Cameron and Heckman (1993) indicate, level of education appears to be a mixed-signal. The authors may want to refer to other studies such as Agarwal and Mazumder (2013) regarding how cognitive skills are measured.

This comment is critical and valuable. In the first draft, we mentioned about "years of education" (i.e., ISCED in particular) as a simple proxy for cognitive skills. Yet, it is true that education cannot cover those skills fully, and the years of education can also relate to non-cognitive skills, as suggested by the referee. First, we note that our aim is not to identify the "cognitive skills" and contrast it with the non-cognitive skills in the employment integration of immigrants. We only explore the heterogeneity of integration outcomes by the non-cognitive skills and how these skills "interact" with standard human capital measures in this process. The "cognitive skill" terminology used in the previous draft was misleading. We now clarified the text by simply using the terminology of "years of education" and referring to this as the "standard human capital measure". Indeed, the literature exceptionally uses education (and some related measures such as training etc.) as the standard measure of human capital. These measures are closer to "cognitive" aspects of human capital. In the paper, we consider education and non-cognitive skills as a part of a more extensive set of human capital. We have clarified the text as mentioned (changes are highlighted with bold text).

(4) This paper uses SOEP 1984-2013. I was wondering why more recent waves are not used.

The reason is practical. First, we have consistent data for the main sample and ancillary (confidential local data) only until 2013. Second, there is also a refreshment sample for immigrants in the year 2013 (a new sample of immigrants). Thus, we cannot use them in our panel data analysis as they are observed only once, and their non-cognitive skills are measured only once or twice in later years (only in 2017). Thus, we keep our sample between 1984 and 2013. We have mentioned the data availability and selection criteria in the text and Footnote 6.

(5) SOEP has other measures of non-cognitive skills such as locus of control. See, for example, Caliendo et al. (2022). The authors may want to consider this measure.

We thank the referee for this point. The paper's main aim is to demonstrate the relationship between the non-cognitive skills and relative labour market outcomes of immigrants. We do not aim to capture all aspects of the non-cognitive skills. Yet, we believe that the paper makes the point with Big-5 as these personality characteristics are sufficient measures of a general set of non-cognitive skills and help to make the point that non-cognitive skills matter in the labour market performance of immigrants, as successfully demonstrated in the employment outcomes of immigrants. Also, adding alternative non-cognitive skill measures such as the locus of control or reciprocity norms requires a deeper discussion of how these characteristics might have related to the labour market performance of the immigrants. Overall, this would require a new structure or a completely new paper. We leave this also for future research. We have mentioned about this issue in Footnote 2.

References

Agarwal, S. and B. Mazumder (2013). Cognitive abilities and household financial decision making. American Economic Journal: Applied Economics 5, 193–207.

Caliendo, M., Cobb-Clark, D.A.A, Obst, C., Seitz, H., and Uhlendorff, A. (2022). Locus of Control and Investment in Training. Journal of Human Resources, forthcoming.

Cameron, Stephen V., and Heckman, James J. 1993. The Nonequivalence of High School Equivalents. Journal of Labor Economics 11, 1-47.

Heckman, James J., and Rubinstein, Yona 2001. The Importance of Non-cognitive Skills: Lessons from the GED Testing Program. American Economic Review 91, 145-149.

6. PLOS authors have the option to publish the peer review history of their article (what does this mean?). If published, this will include your full peer review and any attached files.

If you choose "no", your identity will remain anonymous but your review may still be made public.

Do you want your identity to be public for this peer review? For information about this choice, including consent withdrawal, please see our Privacy Policy.

Reviewer #1: No
